# Comparative evaluation of Biofire Joint Infection Panel, Sepsitest 16S/18S rDNA PCR, and culture to identify microorganisms in explanted heart valves

**DOI:** 10.1128/spectrum.01263-25

**Published:** 2025-07-22

**Authors:** Benjamin Berinson, Konstantin Tanida, Manuel Wolters, Flaminia Olearo, Christoph Sinning, Martin Christner, Martin Aepfelbacher, Holger Rohde

**Affiliations:** 1 Institute for Medical Microbiology, Virology and Hygiene, University Medical Center Hamburg-Eppendorf 37734, Hamburg, Germany; 2 Department for Cardiology, University Medical Center Hamburg-Eppendorf 37734, Hamburg, Germany; University of Maryland School of Medicine, Baltimore, Maryland, USA

**Keywords:** infectious endocarditis, pathogen detection, culture, molecular assays, 16S rDNA PCR, Biofire FilmArray JI panel

## Abstract

Infective endocarditis (IE) is a major life-threatening disease. However, the inability to identify the causative pathogen using conventional microbiological cultures jeopardizes the choice of optimal antibiotic therapy. Culture-independent molecular techniques for pathogen detection may overcome this challenge. Here, two molecular tests (16S/18S rDNA assay [SepsiTest, Molzym, Germany]; Biofire Joint Infection Panel [BJP, bioMérieux, Marcy l’Étoile, France]) were analyzed for their ability to detect pathogens in a series of 100 explanted heart valves. In total, at least one pathogen was detected in 67/100 specimens by one or more methods, and 33/100 specimens were negative in all three assays. Overall, valve-culture had an inferior sensitivity (39.4%), compared to 16S/18S rDNA PCR (90.9%) and the BJP (83.1% [all organisms], 98.2% [on-panel organisms only]). In culture-negative cases, 35 and 32 additional pathogens were identified by 16S/18S rDNA PCR and BJP, respectively. In five culture- and 16S/18S rDNA assay-negative cases, BJP identified correctly additional pathogens. False-positive results were observed in one (16S/18S rDNA PCR) and two (BJP) cases, respectively. In conclusion, both culture-independent assays proved to be reliable and increased the diagnostic yield compared to culture. In general, false negatives (i.e., off-panel pathogens in BJP) and false positives require careful clinical evaluation of results from culture-independent techniques. Diagnostic stewardship is essential to guide the optimal use of molecular techniques in the work-up of infective endocarditis.

**IMPORTANCE:**

Infective endocarditis (IE) therapy must be guided by the identification of the causative pathogen. Microbiological cultures, however, remain negative in a significant number of cases, jeopardizing the choice of optimal anti-infective treatment. This study evaluated the ability of two independent molecular tests (16/18S rDNA PCR, syndromic panel PCR [Biofire JI panel]) to overcome limitations of conventional microbiological approaches to detect microorganisms from explanted heart valve tissues. Both assays proved to have a greater sensitivity compared to conventional culture, and PCR and BJP identified a pathogen in 35 and 32 out of 74 culture negative IE cases, additional pathogens, respectively. The study highlights the great importance of culture-independent approaches to pathogen detection in IE and guides the choice of the optimal assay using diagnostic stewardship interventions.

## INTRODUCTION

Infective endocarditis (IE) poses a major public health challenge with high morbidity and mortality, with an incidence in 2019 of 13.8 cases per 100,000 patients per year (1). Identification of the IE causing pathogen is key to optimal therapy. The current gold standard to detect causative microorganisms is thorough blood culture (BC) diagnostics (2, 3). Nevertheless, culturing the causative pathogen can be challenging, resulting in up to 40% of etiologically unresolved, blood culture-negative IE (BCNIE) cases (4). BCNIE are related to fastidious or non-cultivable organisms, like *Coxiella species*, *Bartonella specie*s, or *Tropheryma whipplei*. For identification of these organisms, serology, as well as species-specific PCR approaches, can be applied (5). Another major cause for BCNIE is antibiotic exposure prior to BC being drawn (6–8). This holds true not only for blood culture diagnostics, but as well heart valve cultures, where the sensitivity of culture-based diagnostic approaches might be even lower (9).

To overcome the challenges of BCNIE and valve culture-negative IE, several new, culture-independent approaches have been employed in recent years, including 16S/18S rDNA PCR followed by amplicon sequencing (3, 5, 10–12). This approach can formally detect all bacteria and fungi, including rare and non-cultivable pathogens. In contrast, syndromic panel PCR (spPCR) assays have a limited pathogen coverage, i.e., are restricted to detect pathogens represented on the panel. This disadvantage is at least partially outweighed by greater sensitivity, faster time to result, and the ability to predict antibiotic resistance profiles by including molecular resistance determinants (12, 13). These culture-independent approaches are also represented in the 2023 Duke Criteria (4) and 2023 ESC Guidelines for IE (2), which now for the first time state that identifying a microorganism on a valve, implantable devices, or an arterial embolus by nucleic-acid-based tests classifies as definite IE (2, 4).

The BioFire Joint Infection Panel (BJP) (bioMérieux, Marcy l’Étoile, France) identifies 31 bacterial and fungal pathogens as well as 10 clinically relevant genetic resistance markers, including *mecA/C*, *vanA/B*, carbapenemase-encoding genes, and *bla*_CTX-M_. Here, we compared the analytical performance of the BJP and a CE-IVD labeled 16S/18S rDNA PCR assay (SepsiTest, UMD SelectNA, Molzym, Germany) to detect microorganisms in 100 explanted heart valves compared with standard culture.

## MATERIALS AND METHODS

### Study setting and inclusion criteria

This prospective single-center study was conducted at the University Medical Center Hamburg-Eppendorf, Germany, a 1,700-bed tertiary care university hospital. The study was performed between April 2023 and December 2024. All surgically explanted heart valves, which were sent for standard of care microbiological diagnostics (described below), were subjected to BJP analysis. Exclusion criteria for BJP analysis were removal of the same heart valve within 30 days and patient age <18 years. In total, 100 samples from 97 patients were included. Of note, three patients had either different heart valves removed or the same heart valve removed more than 30 days apart and are, therefore, considered individual cases. Clinical data, like patient characteristics, valve localization, and final diagnosis, were extracted from the electronic patient chart available.

### Standard of care microbiological analysis of explanted heart valves

Heart valves were sent to the microbiology laboratory in a sterile container by pneumatic post. Tissue homogenization was performed using the IKA Ultra Turrax Tube Drive control homogenizer (IKA Werke GmbH & Co. KG, Staufen, Germany) following the manufacturer’s instructions. In brief, 2 mL of sterile 0.9% NaCl was added to a homogenization tube (BMT-20-S-M-Gamma from IKA), containing sterile metal beads, together with the heart valve. After bead beating for 1 min at 6,000 rpm, 50–100 µL of the homogenate was plated onto Columbia sheep blood agar and chocolate agar for cultivation of aerobic bacteria as well as on Schaedler anaerobic agar (all from Oxoid, Basingstoke, UK) for cultivation of anaerobic bacteria. All aerobic incubation was carried out at 37°C and 5% CO_2_ for up to 14 days, anaerobic incubation was performed for up to 7 days. Plates were evaluated for growth after 24 h, 48 h, 7 days, and 14 days. Additionally, 2 mL of thioglycolate broth was inoculated with 500 µL of the homogenized valve tissue. All microorganisms obtained from cultures were subjected to pathogen identification using a Mass Spectrometry Laser desorption/ionization time of flight (Maldi-ToF) instrument (Microflex, Bruker Daltonics, Bremen, Germany). Any spare specimen was frozen first at −20°C and subsequently stored at −80°C for possible further analysis.

Isolates were subjected to susceptibility testing on a VITEK2 instrument (bioMérieux, Marcy l´Étoile, France) using either the VITEK2 AST-N223 (*Enterobacterales*) or the VITEK2 AST-P611 card (staphylococci, enterococci). Agar diffusion was employed to test fastidious organisms (e.g., streptococci) according to EUCAST protocols.

As part of institutional SOC workflows, all heart valve specimens are also subjected to 16S rDNA PCR analysis, following a recently published protocol (14). In brief, 1 mL of homogenized specimen was depleted of human DNA and subsequently bacterial DNA was isolated according to the manufacturer’s protocol. A PCR for amplification of 16S/18S rDNA was run on a LightCycler480 instrument. Amplification and melt curves were analyzed. Samples with a Ct-value below 35 and a *T*_*m*_ between 86°C and 92°C were regarded as positive. PCR amplicons were purified (QIAquick PCR purification kit; Qiagen, Hilden, Germany) and sent for Sanger sequencing. 16S and 18S sequences were analyzed with the web-based SepsiTest-BLAST application (Molzym).

### Biofire Joint Infection Panel analysis

BJP was performed according to the manufacturer’s guidelines. Briefly, manufacturer-provided hydration solution was loaded to the pouch, and 200 µL of the heart valve homogenate was mixed with the provided sample buffer. This mixture was loaded to the pouch, which then was inserted into the instrument, and the assay was run by the system. An overview of genera or families of bacteria and resistance markers represented on BJA in the here utilized version is provided in the supplemental material (Table S1). Biofire JI panel is not approved for the analysis of heart valve tissue, and results were not communicated to the clinician.

### Verification of discrepant results

To verify potentially discrepant results between culture, 16/18S rDNA PCR, and BJP analysis, respective specimens were subjected to a rerun on the Biofire instrument and, if possible, species-specific PCRs were performed. Primers and probes were selected based on previously published protocols, and sequences are listed in Table S2 (*Escherichia coli* and *S. aureus* [15], *bla_CTX-M_* [16]). For species-specific PCRs, nucleic acids were extracted from homogenized specimens using a MagNa Pure 96 System (Roche, Mannheim, Germany), and PCR was performed on a LightCycler 480 II instrument (Roche, Mannheim, Germany).

### Calculation of diagnostic accuracy parameters

For calculation of diagnostic accuracy parameters, a composite reference standard was defined. A true positive specimen was assumed if a microorganism was identified in at least two different assays. In addition, a true positive result was assumed if a microorganism was identified by one of the employed methods in combination with (1) a concordant result obtained by a species-specific real-time PCR from valve tissue, (2) identification of the identical pathogen from blood cultures drawn during IE treatment at our hospital, or (3) detection of a concordant pathogen in blood cultures drawn in relation to the IE episode, but outside of our institution. If a specimen was negative in all three tests, the result was deemed true negative. Following these definitions, sensitivity and specificity were independently calculated for all three methods.

### Quality control

The quality control (QC) for the MALDI-ToF analysis was performed on a daily basis with *E. coli* ATCC 25922 and *C. albicans* ATCC 25922. The EUCAST and VITEK2 quality control procedure was performed regularly once per week, as previously described (17). Full process control for the species-specific PCRs was performed by an internal spike-in control, which was added during DNA extraction (Cobas omni optimization reagent, Roche). Additionally, the BJP assay QC was performed once with the supplied positive and negative QC test vials.

## RESULTS

### Patient characteristics, assay results, and concordance

In total, 100 explanted heart valve tissue obtained from 97 individual patients between 04/2023 and 12/2024 were investigated. An overview of patient characteristics is given in Table 1. The term “culture” hereafter refers exclusively to valve culture, not blood culture (BC).

**TABLE 1:** Basic patient characteristics

Variable	Patients (*N* = 100)
Sex, male (%)	71 (71%)
Age years, mean (SD; 95% CI)	61.95 (13.39; 59.29–64.6)
Clinical IE diagnosis	78 (78%)
Prosthetic valve, *N* (%)	30 (30%)
Native valve, *N* (%)	70 (70%)
Aortic valve, *N* (%)	57 (57%)
Mitral valve, *N* (%)	35 (35%)
Tricuspic valve, *N* (%)	6 (6%)
Pulmonary valve, *N* (%)	2 (2%)

In total, at least one pathogen was detected in 67/100 specimens by one or more methods (all per specimen results are given in Table S3) and 33/100 specimens were negative in all three assays. Specifically, culture resulted in the detection of at least one pathogen in 26 cases, while 16S/18S rDNA PCR and BJP were positive in 60 and 56 cases, respectively.

In 25/26 (96.2%) of culture-positive cases, 16S/18S rDNA PCR analysis revealed concordant results (Table 2). Case 35 showed growth of *Staphylococcus epidermidis* on solid agar media, whereas 16S rDNA PCR and BJP were negative (Table 2). BJP identified species consistent with culture results in 18/26 (69.2%) of culture-positive cases. In all BJP negative cases, culture identified off-panel organisms (*Mycoplasma hominis n* = 1, *Cutibacterium acnes n* = 1, *S. epidermidis n* = 5*, Lactococcus garvieae n* = 1). In one case (#14), in addition to *Streptococcus* spp. (concordant with culture and 16S rDNA result), BJP also identified *Escherichia coli* with a CTX-M enzyme. Neither rerun nor in-house PCRs for *E. coli* and *bla_CTX-M_* could verify this result, which was consequently interpreted as a false positive call (Table 2).

**TABLE 2:** Results of 16S rDNA PCR and BJP results in comparison to culture positive samples

Study number	Culture	16S/18S rDNA PCR	BJP
4	*Streptococcus agalactiae*	*S. agalactiae*	*S. agalactiae*
6	*Mycoplasma hominis*	*M. hominis*	Negative
14^*a*^	*S. sangunis* Group	*S. gordonii*	*Streptococcus* spp., *E. coli* (CTX-M positive)
16	*Cutibacterium acnes*	*C. acnes*	Negative
24	*Staphylococcus epidermidis*	*S. epidermidis/caprae/capitis*	Negative
25	*Enterococcus faecalis*	*E. faecalis*	*E. faecalis*
33	*S. sangunis* Group	*S. sangunis* Group	*Streptococcus spp.*
35^*b*^	*S. epidermidis*	Negative	Negative
37	*S. aureus*	*S. aureus* Complex	*S. aureus*
38	*E. faecalis*	*E. faecalis*	*E. faecalis*
39	*S. equinus/bovis* Group	*S. equinus/bovis* Group	*Streptococcus* spp.
43	*S. aureus*	*S. aureus* Complex	*S. aureus*
44	*E. faecalis*	*E. faecalis*	*E. faecalis*
45	*E. faecalis*	*E. faecalis*	*E. faecalis*
51	*S. aureus*	*S. aureus* Complex	*S. aureus*
54	*Candida albicans*	*C. albicans*	*C. albicans*
58	*S. equinus/bovis* Group	*S. equinus/bovis* Group	*Streptococcus* spp.
66	*S. epidermidis*	*S. epidermidis*	Negative
75	*S. lugdunensis*	*S. lugdunensis*	*S. lugdunensis*
78	*S. epidermidis*	*S. epidermidis*	Negative
81	*S. lugdunensis*	*S. lugdunensis*	*S. lugdunensis*
86	*S. equi*	*S. equi*	*Streptococcus* spp.
91	*S. aureus*	*S. aureus* Complex	*S. aureus*
94	*Lactococcus garvieae*	*L. garvieae/formosensis*	Negative
97	*S. equinus/bovis* Group	*S. equinus/bovis* Group	*Streptococcus* species
99	*S. epidermidis*	*S. epidermidis*	Negative

^*a*^ *E. coli* with *bla*_CTX-M_ could not be verified with rerun and species-specific PCRs and was therefore regarded as a false positive.

^*b*^ Chart review revealed that in this case several blood cultures were positive for *S. epidermidis* before; thus, this case was regarded as 16S rDNA PCR false-negative.

In 74/100 specimens, cultures remained negative. In 35/74 (47.3 %) culture-negative cases, 16S rDNA PCR revealed a pathogen (Table 3). BJP was positive for the identical pathogen compared to 16S rDNA PCR in 31/35 (88.6%) cases. In one case (#19), BJP remained negative, while 16S rDNA PCR identified *S. mitis* Group, which is an on-panel pathogen. In another case (#20), 16S rDNA analysis identified *Corynebacterium kroppenstedtii/pseudokroppenstedtii,* while BJP identified *E. coli*. Chart review showed that several previous blood cultures grew *E. coli*, and consequently, the 16S rDNA PCR result was deemed false-positive. Two other cases (case #65 and #92) showing discordant results between BJP and 16S rDNA PCR were related to off-panel organisms (*C. striatum, n* = 1; *Lactococcus garvieae/formosensis n* = 1) (Table 3).

**TABLE 3:** Summary of 16S rDNA PCR and BJP results in culture negative samples

Study number	Culture	16S rDNA PCR	BJP
2	Negative	*S. sanguinis* Group	*Streptococcus* spp.
3	Negative	*S. mutans*	*Streptococcus* spp.
12	Negative	*S. equinus/bovis* Group	*Streptococcus* spp.
15	Negative	*S. mitis* Group	*Streptococcus* spp.
17	Negative	*S. dysgalactiae*	*Streptococcus* spp.
19^*a*^	Negative	*S. mitis* Group	Negative
20^*b*^	Negative	*Corynebacterium kroppenstedtii/pseudokroppenstedtii*	*E. coli*
23	Negative	*E. faecalis*	*E. faecalis*
27	Negative	*S. mitis* Group	*Streptococcus* spp.
30	Negative	*S. dysgalactiae*	*Streptococcus* spp.
31	Negative	*S. mitis* Group	*Streptococcus* spp.
34	Negative	*S. agalactiae*	*S. agalactiae*
40	Negative	*S. aureus* Complex	*S. aureus*
42	Negative	*S. aureus* Complex	*S. aureus*
46	Negative	*S. anginosus* Group	*Streptococcus* spp.
56	Negative	*S. aureus* Complex	*S. aureus*
57	Negative	*S. mitis* Group	*Streptococcus* spp.
59	Negative	*S. mitis* Group	*Streptococcus* spp.
60	Negative	*S. mitis* Group	*Streptococcus* spp.
63	Negative	*S. aureus* Complex	*S. aureus*
64	Negative	*S. sangunis* Group	*Streptococcus* spp.
65^*c*^	Negative	*L. garvieae/formosensis*	Negative
67	Negative	*S. pneumoniae*	*S. pneumoniae*
71	Negative	*S. aureus* Complex	*S. aureus*
74	Negative	*S. sanguinis* Group	*Streptococcus* spp.
76	Negative	*E. faecalis*	*E. faecalis*
77	Negative	*S. aureus* Complex	*S. aureus*
79	Negative	*S. aureus* Complex	*S. aureus*
80	Negative	*S. mitis* Group	*Streptococcus* spp.
82	Negative	*S. mutans* Group	*Streptococcus* spp.
88	Negative	*S. sangunis* Group	*Streptococcus* spp.
90	Negative	*S. agalactiae*	*S. agalactiae*
92^*c*^	Negative	*C. striatum*	Negative
93	Negative	*S. equinus/bovis* Group	*Streptococcus* spp.
100	Negative	*S. mitis* Group	*Streptococcus* spp.

^*a*^ Negative BJP result, although on-panel pathogen. Chart review showed several external BC positive for identified pathogen.

^*b*^ Discrepant species ID in BJP and 16S rDNA assay, which was ruled in favor of BJP by the clinician based on previous BC results with *E. coli*.

^*c*^ *Lactococcus garvieae/formosensis* and *C. striatum* were regarded as a true positive result, as same corresponding species were recovered from BC taken from the respective patients. Species are BJP off-panel pathogens.

BJP was positive in six cases negative for culture and 16S rDNA PCR (*S. aureus, n* = 2; *Enterococcus faecalis*, *n* = 2; *S. pneumoniae*, *n* = 1; *E. coli*, *n* = 1) (Table 4). All additional findings obtained by BJP, apart from the *E. coli* call, were confirmed by rerun and/or also previous lab findings and/or chart review of consistent clinical evidence (i.e., external BC results). The identification of *E. coli* could neither be repeated by rerun nor a species-specific PCR and was, therefore, interpreted as a false positive result.

**TABLE 4:** BJP results in culture- and 16S rDNA PCR-negative samples

Study number	Culture	16S rDNA PCR	BJP
1^*a*^	Negative	Negative	*S. aureus*
7^*a*^	Negative	Negative	*E. faecalis*
28^*a*^	Negative	Negative	*E. faecalis*
55^*b*^	Negative	Negative	*E. coli*
62^*c*^	Negative	Negative	*S. aureus*
73^*a*^	Negative	Negative	*S. penumoniae*

^*a*^ Chart review showed previous BC positive for identified pathogen.

^*b*^ *E. coli* identification of BJP could not be reproduced neither by re-run nor using species-specific PCR and was interpreted as a false positive call by BJP.

^*c*^ *S. aureus* was identified via species-specific PCR.

### Diagnostic accuracy of culture, 16S/18S rDNA, and BJP assay

According to the predefined composite reference standard definition for positivity, 66 specimens were true-positives and 34 were true-negatives. In 26 out of 66 true-positive specimens (39.4%), culture identified a pathogen. All isolates were recovered from direct specimen plating; enrichment broth cultures did not yield additional isolates. 16S rDNA PCR identified true-positive samples in 60/66 (90.9%) specimens, and BJP identified true-positive samples in 54/66 cases (83.1%). Culture did not show false-positive results, but in total, resulted in 40 false-negative cases. For 16S rDNA and BJP, 1 and 2 false-positive results were identified, respectively, as well as 6 and 11 false-negative results, respectively. Calculated from these results, sensitivity for valve culture was 39.4% (95% CI 27.6%–52.2%) with a specificity of 100% (95% CI 89.7%–100%). For 16S/18S rDNA, calculated sensitivity was 90.9% (95% CI 81.3%–96.6%) and a specificity of 97.1% (95% CI 84.7%–99.9%), while BJP sensitivity was 83.1% (95% CI 71.7%–91.2%) and a specificity of 94.3% (95% CI 80.8%–99.3%). The pathogen coverage rate of BJP was 84.8% (in 10 cases, off-panel organisms were detected). After removing cases caused by off-panel organisms, BJP sensitivity increased to 98.2% (95% CI 90.3%–100.0%).

### Lab work-flow assessment

The total turnaround time (TTAT), which we defined as the time between sampling and the corresponding test result (i.e., culture and/or 16S rDNA PCR result), was evaluated. For the limited number of culture-positive samples, the mean TTAT to species identification was 81:15 h (median 52:54 h, SD 71.3 h). For 16S rDNA analysis, the TTAT was 100:46 h (median: 92:50 h, SD 69.27 h). This results in a longer TTAT (19:31 h) compared to culture and is a labor-intensive approach. The time to result for BJP is approximately 1 h with a short hands-on time, but the TTAT was not evaluated as these results were not reported to the clinician.

## DISCUSSION

Identifying the causative pathogen is key for optimal antibiotic therapy and critical for management of patients with IE. Therefore, BCNIE and valve culture-negative endocarditis is posing a major challenge for microbiological laboratories and clinicians alike (4, 6–9). Culture-independent approaches hold great promise for overcoming the limitations of traditional culture-based approaches to pathogen detection, and the availability of fully automated spPCR assays opens the way for widespread implementation of amplification-based techniques outside of dedicated, specialized molecular biology laboratories. Here, we investigated the analytical performance of a recently developed spPCR, the BJP, marketed for the detection of bacterial pathogens in synovial fluid, to identify pathogens in explanted heart valve specimens. Results were compared with standard culture and a commercial 16S/18S rDNA PCR assay (SepsiTest, UMD SelectNA, Molzym). Both molecular assays demonstrated superior sensitivity (16S/18S rDNA: 90.9%; BJP: 83.1%) to detect pathogens compared to culture alone (39.4%). These results strongly support the incorporation of molecular techniques into standard protocols for the analysis of explanted heart valves in suspected cases of IE.

Homogenized heart valve tissue can pose a critical challenge to the performance of fully integrated PCR assays, for example, due to the presence of inhibitors or capillary blockage. The decision to use the BJP assay was based on our own and published experience with the BJP PCR (18–22), which demonstrated robust performance even in a cell debris-rich specimen such as synovial fluid from patients with bacterial arthritis. However, it must be acknowledged that others have used the Biofire BCID1 panel to detect pathogens in explanted heart valves (23), suggesting that other fully integrated PCR assays may work.

A major limitation of using spPCR approaches is the potential presence of off-panel organisms. In our series, 10 cases were caused by organisms not represented on the BJP, resulting in an overall lower sensitivity compared to 16S/18S rDNA PCR. In fact, the sensitivity of the BJP for on-panel organisms was 98.2%, which is higher than 16S/18S rDNA PCR, indicating the robust analytical performance in on-panel cases. Nevertheless, the inability to detect CoNS and *Cutibacterium* spp. is critical, and indeed in this cohort, six cases caused by the aforementioned off-panel organisms resulted in false-negative calls. Other spPCR assays, e.g., Biofire BCID2 or the Unyvero blood culture assay, have a built-in capability to detect CoNS, and the Unyvero assay even detects *Cutibacterium* spp. (17, 24). Future comparative studies will, therefore, need to address the question of whether the broader coverage of pathogens in some cases outweighs potential drawbacks. BCID2 and the Unyvero system have been validated on positive blood culture bottles and, therefore, start from a higher bacterial load than directly from primary specimens (i.e., heart valves), and the assay design could lead to false-negative results in samples with pathogen counts below the detection level.

A major advantage of the BJP assay is its ease of use and rapid TTAT. While TTAT for BJP was not evaluated here, a recent study from our laboratory showed that BJP data were available after approximately 14 h for joint fluids, which follow a similar workflow in our laboratory as heart valves (22). In contrast, the TTAT of 16S/18S rDNA PCR in the present study was around 100 h. Furthermore, 16S/18S rDNA analysis requires significant hands-on time (e.g., sample preparation, DNA extraction, PCR, sequencing, manual analysis of sequencing data), which highlights the advantages of the BJP assay as a first-line molecular test in the IE scenario. In addition, 16S/18S rDNA PCR does not allow prediction of antibiotic susceptibility, whereas the BJP has a built-in ability to detect 10 relevant resistance markers. In particular, the detection of *mecA/C* can be critical in IE patients, and in some reports, MRSA caused 7.6%–13.8% (depending on the study population) (25).

Interestingly, molecular testing identified streptococci in 24/74 culture-negative cases. While acquired resistance in beta-hemolytic streptococci is not clinically relevant, changes in penicillin susceptibility, particularly in viridans streptococci, associated with altered penicillin-binding proteins are a major concern and relevant to the management of IE. At present, due to the complexity of the underlying genomic rearrangements, PCR assays to predict penicillin susceptibility or even MICs do not exist. In the future, it seems possible that metagenomic sequencing will overcome these current analytical limitations (12, 26).

As molecular testing generally involves significant additional costs, it is imperative that it be closely integrated into diagnostic stewardship programs. A recent study in our institute found that in 43.3% of 687 cases in which 16S rDNA PCR was performed on explanted heart valves, a pathogen was already identified from blood cultures, i.e., molecular analysis had little or no impact on clinical management (27). Therefore, a diagnostic stewardship framework is necessary to guide decisions when to use molecular techniques. We propose to use the BJP only in suspected IE cases in which valve cultures and BC are negative. If the BJP is negative, a 16S rDNA PCR should be added as an unbiased approach.

This study has several limitations, i.e., the monocentric study design, the limited sample size, and, consequently, the limited range of species. Therefore, it is not possible to extrapolate performance to other less commonly encountered pathogens. Notably, no resistant isolates were identified in this cohort, preventing an evaluation of the BJP’s performance in detecting resistance. Another limitation is the use of a composite reference standard. This could have led to an incorporation bias and overestimation of assay performance, and consequently, sensitivity and specificity for 16S/18S PCR and BJP may appear better than they truly are. In addition, a spectrum bias may have been introduced, i.e., the composite reference standard may favor pathogens more likely to be detected by multiple assays or those covered by species-specific PCRs or blood culture protocols. Rare or off-panel organisms (especially those not detected by BJP) may be misclassified as false negatives, underestimating their detection rate. The results from BJP were not communicated to the clinician, and therefore, the impact on patient management was not investigated.

In conclusion, we provide evidence that 16S/18S rDNA PCR and BJP have superior sensitivity over valve culture, supporting their use, e.g., in pre-treated patients. For on-panel organisms, BJP sensitivity was higher compared to 16S/18S rDNA PCR analysis, while 16S/18S rDNA PCR showed broader pathogen coverage. To combine the specific strengths of both assays, we suggest their use as part of a staged diagnostic approach. Future studies are warranted to elucidate the usefulness of culture-independent approaches for the diagnosis of IE in a real-life setting.

## AFTER EPUB

[This article was published on 22 July 2025 with an omitted line in the Acknowledgments. The Acknowledgments was corrected in the current version, posted on 31 July 2025.]

## SUPPLEMENTAL MATERIAL

Table S1 spectrum.01263-25-s0001.docx Spectrum of species and resistance determinants detected by the Biofire JI panel.

Table S2 spectrum.01263-25-s0002.xlsx Primers used in this study.

Table S3 spectrum.01263-25-s0003.docx Overview of results for valve culture, 16S/18S rDNA analysis.

ASM does not own the copyrights to Supplemental Material that may be linked to, or accessed through, an article. The authors have granted ASM a non-exclusive, world-wide license to publish the Supplemental Material files. Please contact the corresponding author directly for reuse.
