# Supplementary material for: Comparative evaluation of Biofire Joint Infection Panel, Sepsitest 16S/18S rDNA PCR, and culture to identify microorganisms in explanted heart valves
Source: Microbiol Spectr. 2025 Jul 22;13(9):e01263-25. doi: 10.1128/spectrum.01263-25 (PMC12403625; doi:10.1128/spectrum.01263-25)
Supplement: Table S1 — Spectrum of species and resistance determinants detected by the Biofire JI panel. [file spectrum.01263-25-s0001.docx]

| **Supplementary table 1: Spectrum of species and resistance determinants detected by the Biofire JI panel** | | |  |
| --- | --- | --- | --- |
| **GRAM-POSITIVE BACTERIA** | **GRAM-NEGATIVE BACTERIA** | **YEAST** | **ANTIMICROBIAL RESISTANCE GENES** |
| *Anaerococcus prevotii/vaginalis* |  |  |  |
| *Clostridium perfringens* | *Bacteroides fragilis* | *Candida*spp. | **Carbapenemases** |
| *Cutibacterium avidum/granulosum* | *Citrobacter* | *Candida albicans* | IMP |
| *Enterococcus faecalis* | *Enterobacter cloacae* complex |  | KPC |
| *Enterococcus faecium* | *Escherichia coli* |  | NDM |
| *Finegoldia magna* | *Haemophilus influenzae* |  | Oxa-48-like |
| *Parvimonas micra* | *Kingella kingae* |  | VIM |
| *Peptoniphilus* | *Klebsiella aerogenes* |  | **ESBL** |
| *Peptostreptococcus anaerobius* | *Klebsiella pneumoniae* group |  | CTX-M |
| *Staphylococcus aureus* | *Morganella morganii* |  | **Methicillin Resistance** |
| *Staphylococcus lugdunensis* | *Neisseria gonorrhoeae* |  | *mecA/C* and MREJ (MRSA) |
| *Streptococcus* spp*.* | *Proteus* spp*.* |  | **Vancomycin Resistance** |
| *Streptococcus agalactiae* | *Pseudomonas aeruginosa* |  | *vanA/B* |
| *Streptococcus pneumoniae* | *Salmonella* spp*.* |  |  |
| *Streptococcus pyogenes* | *Serratia marcescens* |  |  |
