# Supplementary material for: Comparative evaluation of Biofire Joint Infection Panel, Sepsitest 16S/18S rDNA PCR, and culture to identify microorganisms in explanted heart valves
Source: Microbiol Spectr. 2025 Jul 22;13(9):e01263-25. doi: 10.1128/spectrum.01263-25 (PMC12403625; doi:10.1128/spectrum.01263-25)
Supplement: Table S3 — Overview of results for valve culture, 16S/18S rDNA analysis. [file spectrum.01263-25-s0003.docx]

**Suppl table 3 Overview of results for valve culture, 16S/18S rDNA analysis and BJP results, as well as valve locations and type**

| Study number | Result via culture | Result via 16S/18S rDNA sequencing | Result via BJP | Valve | type of valve (native/prosthetic) |
| --- | --- | --- | --- | --- | --- |
| 1 | none | none | *Staphylococcus aureus* | tricuspid valve | native |
| 2 | none | *Streptococcus sanguinis Group* | *Streptococcus spp.* | aortic valve | native |
| 3 | none | *S. mutans* | *Streptococcus spp.* | aortic valve | native |
| 4 | *S. agalactiae* | *S. agalactiae* | *S. agalactiae* | mitral valve | native |
| 5 | none | none | none | aortic valve | native |
| 6 | *Mycoplasma hominis* | *M. hominis* | none | aortic valve | prosthetic |
| 7 | none | none | *Enterococcus faecalis* | aortic valve | prosthetic |
| 8 | none | none | none | aortic valve | prosthetic |
| 9 | none | none | none | aortic valve | prosthetic |
| 10 | none | none | none | tricuspid valve | native |
| 11 | none | none | none | aortic valve | prosthetic |
| 12 | none | *S. equinus/bovis* Group | *Streptococcus spp.* | mitral valve | prosthetic |
| 13 | none | none | none | aortic valve | prosthetic |
| 14 | *S. sangunis* Group | *S. gordonii* | *Streptococcus spp*., *Escherichia coli* with CTXM | aortic valve | prosthetic |
| 15 | none | *S. myci* | *Streptococcus spp.* | mitral valve | native |
| 16 | *Cutibacterium acnes* | *C. acnes* | none | aortic valve | prosthetic |
| 17 | none | *S. dysgalactiae* | *Streptococcus spp.* | aortic valve | prosthetic |
| 18 | none | none | none | mitral valve | native |
| 19 | none | *S. mitis* Group | none | aortic valve | native |
| 20 | none | *C. kroppenstedtii/pseudokroppenstedtii* | *E. coli* | aortic valve | prosthetic |
| 21 | none | none | none | aortic valve | prosthetic |
| 22 | none | none | none | aortic valve | native |
| 23 | none | *E. faecalis* | *E. faecalis* | aortic valve | prosthetic |
| 24 | *S. epidermidis* | *S. epidermidis/caprae/capitis* | none | aortic valve | native |
| 25 | *E. faecalis* | *E. faecalis* | *E. faecalis* | aortic valve | prosthetic |
| 26 | none | none | none | aortic valve | native |
| 27 | none | *S. mitis* Group | *Streptococcus spp.* | aortic valve | native |
| 28 | none | none | *E. faecalis* | aortic valve | prosthetic |
| 29 | none | none | none | mitral valve | native |
| 30 | none | *S. dysgalactiae* | *Streptococcus spp.* | mitral valve | native |
| 31 | none | *S. mitis* Group | *Streptococcus spp.* | aortic valve | prosthetic |
| 32 | none | none | none | mitral valve | native |
| 33 | *S. sangunis* Group | *S. sangunis* Group | *Streptococcus spp.* | mitral valve | native |
| 34 | none | *S. agalactiae* | *S. agalactiae* | aortic valve | native |
| 35 | *S. epidermidis* | none | none | mitral valve | prosthetic |
| 36 | none | none | none | aortic valve | native |
| 37 | *S. aureus* | *S. aureus* Complex | *S. aureus* | aortic valve | native |
| 38 | *E. faecalis* | *E. faecalis* | *E. faecalis* | aortic valve | native |
| 39 | *S. equinus/bovis* Group | *S. equinus/bovis* Group | *Streptococcus spp.* | aortic valve | native |
| 40 | none | *S. aureus* Complex | *S. aureus* | mitral valve | native |
| 41 | none | none | none | aortic valve | native |
| 42 | none | *S. aureus* Complex | *S. aureus* | tricuspid valve | native |
| 43 | *S. aureus* | *S. aureus* Complex | *S. aureus* | mitral valve | native |
| 44 | *E. faecalis* | *E. faecalis* | *E. faecalis* | mitral valve | native |
| 45 | *E. faecalis* | *E. faecalis* | *E. faecalis* | aortic valve | native |
| 46 | none | *Streptococcus anginosus* Group | *Streptococcus spp.* | mitral valve | native |
| 47 | none | none | none | mitral valve | native |
| 48 | none | none | none | aortic valve | native |
| 49 | none | none | none | mitral valve | native |
| 50 | none | none | none | aortic valve | native |
| 51 | *S. aureus* | *S. aureus* | *S. aureus* | mitral valve | native |
| 52 | none | none | none | aortic valve | native |
| 53 | none | none | none | aortic valve | prosthetic |
| 54 | *Candida albicans* | *C. albicans* | *C. albicans* | pulmonary valve | native |
| 55 | none | none | *E. coli* | aortic valve | native |
| 56 | none | *S. aureus* Complex | *S. aureus* | aortic valve | prosthetic |
| 57 | none | *S. mitis* Group | *Streptococcus spp.* | mitral valve | native |
| 58 | *S. equinus/bovis* Group | *S. equinus/bovis* Group | *Streptococcus spp.* | aortic valve | native |
| 59 | none | *S. mitis* Group | *Streptococcus spp.* | aortic valve | prosthetic |
| 60 | none | *S. mitis* Group | *Streptococcus spp.* | mitral valve | native |
| 61 | none | none | none | mitral valve | prosthetic |
| 62 | none | none | *S. aureus* | aortic valve | native |
| 63 | none | *S. aureus* Complex | *S. aureus* | aortic valve | native |
| 64 | none | *S. sangunis* Group | *Streptococcus spp.* | mitral valve | native |
| 65 | none | *Lactococcus garvieae/formosensis* | none | aortic valve | prosthetic |
| 66 | *S.* *epidermidis* | *S. epidermidis* | none | mitral valve | prosthetic |
| 67 | none | *S. pneumoniae* | *S. pneumoniae* | aortic valve | native |
| 68 | none | none | none | aortic valve | native |
| 69 | none | none | none | mitral valve | native |
| 70 | none | none | none | aortic valve | native |
| 71 | none | *S. aureus* Complex | *S. aureus* | mitral valve | native |
| 72 | none | none | none | aortic valve | native |
| 73 | none | none | *S. penumoniae* | mitral valve | native |
| 74 | none | *S. sanguinis Group* | *Streptococcus spp.* | mitral valve | native |
| 75 | *S. lugdunensis* | *Staphylococcus lugdunensis* | *S. lugdunensis* | mitral valve | native |
| 76 | none | *E. faecalis* | *E. faecalis* | aortic valve | native |
| 77 | none | *S. aureus* Complex | *S. aureus* | pulmonary valve | prosthetic |
| 78 | *S. epidermidis* | *S. epidermidis* | none | aortic valve | native |
| 79 | none | *S. aureus* Complex | S. aureus | tricuspid valve | native |
| 80 | none | *S. mitis* Group | *Streptococcus spp.* | mitral valve | native |
| 81 | S. lugdunensis | *S. lugdunensis* | *S. lugdunensis* | tricuspid valve | native |
| 82 | none | *S. mutans* Group | *Streptococcus spp.* | mitral valve | native |
| 83 | none | none | none | aortic valve | prosthetic |
| 84 | none | none | none | mitral valve | prosthetic |
| 85 | none | none | none | aortic valve | native |
| 86 | *S. equi* | *S. equi* | *Streptococcus spp.* | mitral valve | native |
| 87 | none | none | none | aortic valve | native |
| 88 | none | *S. sangunis* Group | *Streptococcus spp.* | aortic valve | native |
| 89 | none | none | none | mitral valve | native |
| 90 | none | *S. agalactiae* | *S. agalactiae* | aortic valve | prosthetic |
| 91 | *S. aureus* | *S. aureus* Complex | *S. aureus* | aortic valve | native |
| 92 | none | *C. striatum* | none | aortic valve | native |
| 93 | none | *Streptococcus equinus/bovis* Group | *Streptococcus spp.* | mitral valve | native |
| 94 | *L. garvieae* | *L. garvieae/formosensis* | none | tricuspid valve | native |
| 95 | none | none | none | mitral valve | prosthetic |
| 96 | none | none | none | mitral valve | prosthetic |
| 97 | *S. equinus/bovis* Group | *S. equinus/bovis* Group | *Streptococcus spp.* | aortic valve | native |
| 98 | none | none | none | aortic valve | prosthetic |
| 99 | *S. epidermidis* | *S. epidermidis* | none | aortic valve | native |
| 100 | none | *S. mitis* Group | *Streptococcus spp.* | mitral valve | native |
